# OmpK36 deficiency and inducible AmpC β-lactamase synergistically drive imipenem resistance in *Klebsiella aerogenes*

**DOI:** 10.1128/spectrum.04108-25

**Published:** 2026-05-29

**Authors:** Shumi Shang, Liyang Chen, Xiaosi Li, Siqi Xu, Heping Shen, Fuping Hu, Wenting Tang, Xiaoyan Wu

**Affiliations:** 1 Department of Laboratory Medicine, the Second Affiliated Hospital of Jiaxing University 569220, Jiaxing, Zhejiang Province, China; 2 Department of Infectious Disease, the Second Affiliated Hospital of Jiaxing University, Jiaxing, China; 3 Institute of Antibiotics, Huashan Hospital, Fudan University, Shanghai, China; Seton Hall University, South Orange, New Jersey, USA; Indian Institute of Technology Jodhpur, Jodhpur, Rajasthan, India

**Keywords:** *Klebsiella aerogenes*, OmpK36, inducible AmpC β-lactamase, carbapenem resistance

## Abstract

This study systematically elucidates how ST88-KL186 *Klebsiella aerogenes* evolved carbapenem resistance through a dual synergistic mechanism involving porin deficiency and inducible AmpC β-lactamase, providing a mechanistic explanation for carbapenem resistance independent of carbapenemase production. From seven homologous strains (single-nucleotide polymorphism [SNP] difference 3–30) consecutively isolated from a patient with post-pancreaticoduodenectomy infection, three subsequent ascites isolates (R5–R7) developed carbapenem resistance after imipenem treatment. All strains carried the OmpK36 G138S mutation, which reduces pore diameter by ~15%, and displayed an inducible AmpC enzyme. However, an additional Q172Ter truncating mutation in OmpK36 was acquired exclusively in the resistant strains (R5–R7). Molecular dynamics simulations confirmed that the truncated protein loses conformational stability, resulting in a complete loss of functional porin. Although the susceptible strains already had a narrowed pore and inducible AmpC enzyme, residual porin function allowed enough drug entry to maintain susceptibility. In contrast, in resistant strains, loss of OmpK36 served as the primary barrier restricting drug entry, while the small amount of imipenem that entered was sufficient to induce high-level AmpC enzyme expression. The synergistic interplay between these two mechanisms: a “permeability barrier” coupled with “enzymatic hydrolysis”—ultimately overcame the carbapenem resistance threshold. This study underscores the importance of phenotypic screening for inducible AmpC enzymes in clinical practice and provides key evidence for optimizing the diagnosis and management of carbapenem-resistant Gram-negative bacteria.

**IMPORTANCE:**

The synergistic resistance mechanism of OmpK36 deficiency and induced AmpC β-lactamase revealed in this study provides a critical theoretical supplement for understanding carbapenem resistance independent of carbapenemases. Structure-function analysis confirmed that the truncation mutation in OmpK36 in resistant strains leads to premature termination of translation and complete loss of the protein channel, thereby preventing drug entry into the cell while still triggering high expression of AmpC enzymes by the small amount of drug that does enter. The synergistic effect of these two mechanisms confers the carbapenem-resistant phenotype in *Klebsiella aerogenes*. This finding not only broadens our understanding of the evolution of adaptive bacterial resistance but also suggests that clinical microbiology laboratories should incorporate testing for this type of inducible AmpC enzyme into routine protocols, thereby providing direct evidence for precise identification of hidden resistance phenotypes and optimization of treatment regimens for severe infections.

## INTRODUCTION

*Klebsiella aerogenes*, originally named *Enterobacter aerogenes*, is a Gram-negative, facultatively anaerobic bacillus. It was once classified under the genus *Enterobacter* but was later reclassified into the genus *Klebsiella* following genomic analyses confirming its closer phylogenetic relationship to *Klebsiella pneumoniae* (1, 2). *K. aerogenes* can cause various infections, including respiratory tract infections, urinary tract infections, infective endocarditis, skin/soft tissue infections, intra-abdominal infections, and osteomyelitis. Compared to other members of the order *Enterobacterales*, *K. aerogenes* is more likely to lead to septic shock, is associated with poorer patient prognosis, and carries a related mortality rate as high as 13.5% to 28% (3, 4).

*K. aerogenes* is a member of the ESKAPE pathogen family, and its resistance mechanisms primarily include alteration or loss of porins, activation of efflux pumps, production of inactivating enzymes, and alteration of drug target sites (5, 6). Additionally, *K. aerogenes* can acquire resistance genes, such as AmpC β-lactamases, extended-spectrum β-lactamases, and carbapenemases via plasmids (7). Notably, *K. aerogenes* harbors an intrinsic chromosomal *ampC* gene encoding a class C β-lactamase, which cannot be reliably detected by antimicrobial susceptibility testing (8). Furthermore, high-level AmpC enzyme expression can be triggered not only inductively but, more importantly, through the selection of hyperproducing mutants (i.e., derepressed subpopulations) under β-lactam exposure, adding another layer of complexity to its resistance profile (9).

Carbapenem-resistant *Enterobacteriaceae* remain a major challenge in public health. Data from the CHINET Antimicrobial Resistance Surveillance Network indicate no reports of carbapenem-resistant *K. aerogenes* (CRKA) isolates between 2005 and 2008 (10). However, its reporting frequency has increased significantly in recent years. In 2011, the prevalence of CRKA in a Shanghai hospital reached as high as 21% (11). Despite its significant clinical importance, the detection rate of carbapenem-resistant *K. aerogenes* remains relatively low. Research on carbapenem-resistant *K. aerogenes* is still relatively scarce, and its resistance mechanisms have not been systematically elucidated.

In this study, seven *K. aerogenes* strains were consecutively isolated from the same patient, progressively developing a carbapenem-resistant phenotype during treatment. Antimicrobial susceptibility testing and whole-genome sequencing analysis revealed that three CRKA strains acquired carbapenem resistance through non-carbapenemase mechanisms, the synergistic action of an OmpK36 porin truncating mutation and inducible AmpC enzyme-mediated resistance to carbapenems. These findings highlight the intricate evolutionary trajectory of *K. aerogenes* under antibiotic pressure and provide critical insights for the clinical management of such atypical resistant phenotypes.

## RESULTS

### Overview of the *K. aerogenes* clinical isolates

**Fig 1:**
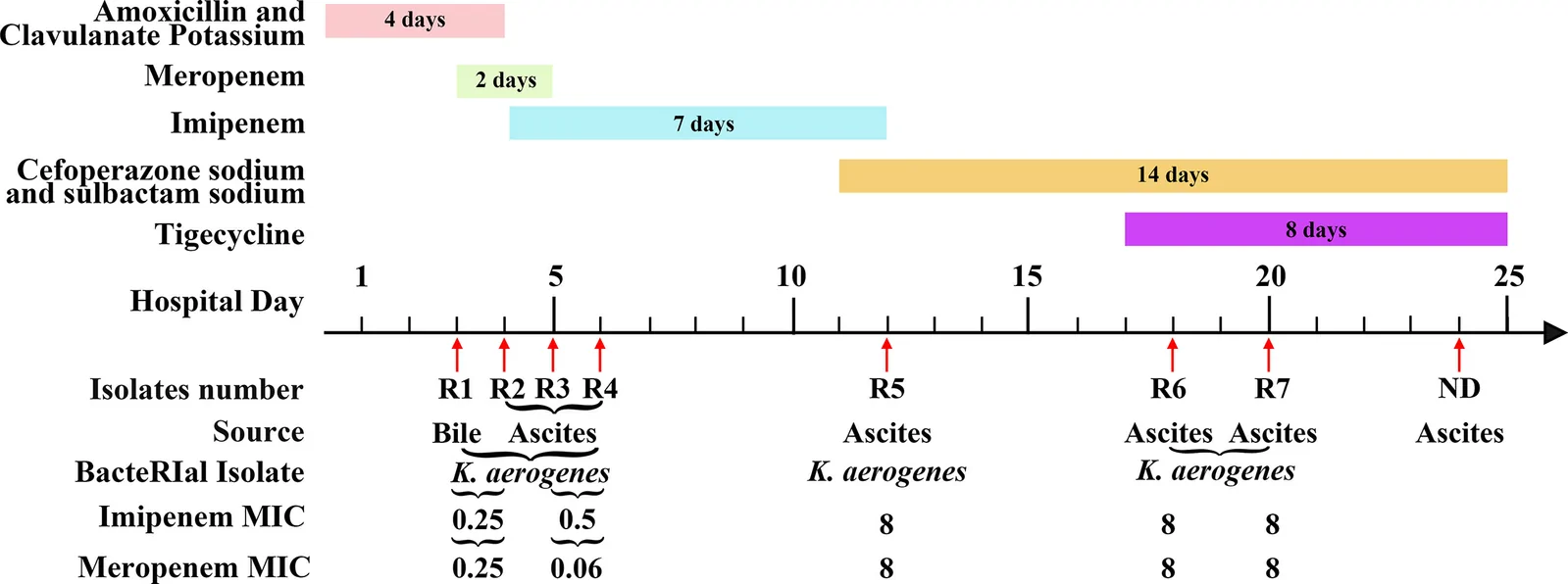
Patient treatment process and sample collection timeline. The bar chart above the timeline represents antibiotic treatment regimens and durations. Information below the timeline includes strain designation, specimen source, and minimum inhibitory concentrations (MICs) for imipenem and meropenem. The day of admission is designated as day 1, with surgery performed on day 2.

*K. aerogenes* strains R1 to R7 were consecutively isolated from a 77-year-old male patient. The patient underwent radical pancreatoduodenectomy at Jiaxing Second Hospital for a gastric space-occupying lesion. Prophylactic antibiotic therapy with amoxicillin-clavulanate (1.2 g, q8h, for 4 days) was initiated 1 day before surgery and continued for 2 days postoperatively, until a bile sample was sent for culture, from which a multidrug-susceptible *K. aerogenes* strain (R1) was isolated. Based on the antimicrobial susceptibility results for strain R1, clinical treatment was switched to meropenem (0.5 g, q8h, 2-day course) and imipenem-cilastatin sodium (0.5 g, q6h, 8-day course). During this period, three additional multidrug-susceptible *K. aerogenes* strains (R2–R4) were consecutively isolated from the patient’s ascites. On postoperative day 10, after isolating carbapenem-resistant *K. aerogenes* from the patient’s ascites, imipenem-cilastatin sodium therapy was discontinued. According to the updated susceptibility profile, the regimen was adjusted to cefoperazone-sulbactam sodium (3 g, q12h, for 6 days), followed by cefoperazone-sulbactam sodium (3 g, q12h, for 8 days) in combination with tigecycline (50 mg, q12h, for 8 days). During this procedure, two carbapenem-resistant *K. aerogenes* strains (R6, R7) were isolated. No pathogens were detected in ascites samples submitted for testing on postoperative day 21. The complete medication timeline for the patient is shown in Fig. 1.

### Antibiotic susceptibility of *K. aerogenes*

Antibiotic susceptibility test results showed (Table 1) that strains R1, R2, R3, and R4 exhibited high susceptibility to carbapenems, cephalosporins, aztreonam, amikacin, cefoperazone-sulbactam, piperacillin-tazobactam, aztreonam-avibactam, and imipenem-relipenimide. Strains R5, R6, and R7 were carbapenemase-resistant *K. aerogenes*, exhibiting high levels of resistance to imipenem, meropenem, ceftriaxone, cefepime, ciprofloxacin, and piperacillin-tazobactam, and cefoperazone-sulbactam. They exhibited susceptibility to the β-lactam combination ceftazidime-avibactam and amikacin-avibactam, but intermediate susceptibility to imipenem-relibactam. The carbapenemase inhibitor enhancement test and colloidal gold assay failed to detect carbapenemase types in strains R5, R6, and R7. Neither did sequencing reveal carbapenemase genes in these three strains. Ceftazidime-avibactam discs were added to the carbapenemase inhibitor-enhancement test plates. Notably, the ceftazidime-avibactam disc exhibited a characteristic “D”-shaped zone on the side facing the imipenem disc (Fig. S1), suggesting that the resistance phenotype of these strains may be mediated by mechanisms other than carbapenemase production.

**TABLE 1:** Antimicrobial susceptibility of all clinical isolates of *K. aerogenes^a^*

Antimicrobial agents	MICs (mg/L)						
	R1	R2	R3	R4	R5	R6	R7
MEM	0.25(S)	0.25(S)	0.06(S)	0.06(S)	8(R)	8(R)	8(R)
IPM	0.25(S)	0.25(S)	0.5(S)	0.5(S)	8(R)	8(R)	8(R)
IMR	0.125/4(S)	0.125/4(S)	0.5/4(S)	0.5/4(S)	2/4(I)	2/4(I)	2/4(I)
CAZ	2(S)	2(S)	0.5(S)	1(S)	2(S)	2(S)	2(S)
CZA	0.5/4(S)	0.125/4(S)	0.25/4(S)	0.25/4(S)	2/4(S)	2/4(S)	2/4(S)
ATM	2(S)	0.5(S)	0.5(S)	0.25(S)	1(S)	1(S)	1(S)
AZA	0.25/4(S)	0.5/4(S)	0.5/4(S)	0.5/4(S)	1/4(S)	1/4(S)	1/4(S)
AMK	2(S)	1(S)	1(S)	2(S)	8(I)	2(S)	2(S)
FEP	0.25(S)	0.25(S)	0.25(S)	0.25(S)	16(R)	16(R)	16(R)
ERV	0.25(S)	0.25(S)	0.25(S)	0.25(S)	0.5(S)	0.5(S)	0.5(S)
CRO	0.25(S)	0.25(S)	0.25(S)	0.25(S)	32(R)	32(R)	32(R)
CIP	0.06(S)	0.06(S)	0.06(S)	0.06(S)	1(R)	1(R)	1(R)
TZP	4/4(S)	4/4(S)	2/4(S)	8/4(S)	64/4(R)	64/4(R)	64/4(R)
TGC	0.5(S)	0.5(S)	0.5(S)	0.5(S)	2(S)	2(S)	2(S)
CSL	≤0.5(S)	≤0.5(S)	≤0.5(S)	≤0.5(S)	16(S)	16(S)	16(S)
COL	0.25(S)	0.25(S)	0.25(S)	0.25(S)	0.25(S)	0.25(S)	0.25(S)

^*a*^ MIC, minimal inhibitory concentration; S, susceptible; I, intermediate; R, resistant; MEM, meropenem; IPM, imipenem; IMR, imipenem-relebactam; CAZ, ceftazidime; CZA, ceftazidime-avibactam; ATM, aztreonam; AZA, aztreonam-avibactam; AMK, amikacin; FEP, cefepime; ERV, eravacycline; CRO, ceftriaxone; CIP, ciprofloxacin; TZP, piperacillin-tazobactam; TGC, tigecycline; COL, colistin.

### Virulence and drug resistance genes in *K. aerogenes*

Analysis of five *K. aerogenes* strains isolated from bile (R1) and ascites (R4, R5, R6, R7) revealed that all strains belonged to the same sequence type ST88 and capsular serotype KL186 (only R7 was O13 type, while the others were O12 type). Genomic single-nucleotide polymorphism (SNP) distances indicated close phylogenetic relationships among the five strains, with particularly high relatedness between R6 and R7 (SNP = 5) and R1 and R4 (SNP = 8). Genetically, all strains carried a complete set of virulence genes, including iron-uptake systems, such as yersiniabactin (*ybtA–ybtX*, *irp1*, *irp2*, *fyuA*), aerobactin (*iucABCD–iutA*), salmochelin (*iroB*, *iroN*), and enterobactin (*entA–entE*, *fepA–fepG*); adhesion-related genes (*fimC*, *fimD*, *yagW/ecpD*); immune evasion-related genes (*ugd*, *rcsB*, *ompA*); and type VI secretion system genes (*hcp/tssD*, *vipB/tssC*). Furthermore, all five *K. aerogenes* strains carried genes and mutations associated with resistance to quinolones (*gyrA*, *gyrB*, *parC*) and fosfomycin (*fosA*), as well as variations in efflux pump regulatory genes (*acrR*, *ramR*) and porin genes (*ompK36*, *ompK37*).

### Effect of efflux pump inhibitors on antimicrobial susceptibility of *K. aerogenes*

To clarify the role of efflux pumps in the antibiotic resistance of *K. aerogenes*, this study treated strains R1–R7 with efflux pump inhibitors carbonyl cyanide 3-chlorophenylhydrazone (CCCP) and PAβN and assessed changes in their antimicrobial susceptibility. The original susceptibility results (Table 1) showed that R5, R6, and R7 exhibited resistance (R) or intermediate (I) activity against imipenem, meropenem, cefepime, ceftriaxone, ciprofloxacin, piperacillin-tazobactam. Following PAβN and CCCP treatment (Tables 2 and 3), although the minimum inhibitory concentration (MIC) values for ceftriaxone decreased in R5, R6, and R7, they remained at resistant levels, indicating that efflux pumps played only a limited role in the resistance mechanisms of these *K. aerogenes* strains.

**TABLE 2:** Effect of CCCP on antimicrobial susceptibility of *K. aerogenes^a^*

Antimicrobial agents	MICs (mg/L)						
	R1-CCCP	R2-CCCP	R3-CCCP	R4-CCCP	R5-CCCP	R6-CCCP	R7-CCCP
MEM	0.125(S)	0.125(S)	0.125(S)	≤0.06(S)	8(R)	8(R)	4(R)
IPM	0.25(S)	0.25(S)	0.5(S)	0.5(S)	8(R)	8(R)	8(R)
IMR	≤0.06/4(S)	≤0.06/4(S)	≤0.06/4(S)	≤0.06/4(S)	1/4(S)	2/4(I)	2/4(I)
CAZ	1(S)	2(S)	2(S)	1(S)	2(S)	2(S)	2(S)
CZA	0.25/4(S)	0.25/4(S)	0.25/4(S)	≤0.06/4(S)	2/4(S)	1/4(S)	1/4(S)
ATM	0.125(S)	≤0.06(S)	0.06(S)	≤0.06(S)	1(S)	1(S)	1(S)
AZA	0.125/4(S)	≤0.06/4(S)	0.06/4(S)	≤0.06/4(S)	1/4(S)	1/4(S)	1/4(S)
AMK	1(S)	1(S)	1(S)	1(S)	4(S)	1(S)	1(S)
FEP	≤0.25(S)	≤0.25(S)	≤0.25(S)	≤0.25(S)	16(R)	16(R)	16(R)
ERV	0.125(S)	0.125(S)	0.125(S)	0.06(S)	0.5(S)	0.125(S)	0.25(S)
CRO	0.25(S)	0.25(S)	0.25(S)	≤0.25(S)	32(R)	8(R)	8(R)
CIP	≤0.06(S)	≤0.06(S)	≤0.06(S)	≤0.25(S)	1(R)	1(R)	1(R)
TZP	2/4(S)	2/4(S)	4/4(S)	≤2/4(S)	64/4(R)	32/4(R)	64/4(R)
TGC	0.5(S)	0.5(S)	0.5(S)	0.5(S)	2(S)	2(S)	2(S)
COL	0.06(S)	0.06(S)	0.125(S)	0.06(S)	0.5(S)	0.06(S)	0.125(S)

^*a*^ R1-CCCP, R2-CCCP, R3-CCCP, R4-CCCP, R5-CCCP, R6-CCCP, and R7-CCCP represent strains treated with the efflux pump inhibitor CCCP. The full names corresponding to the antibiotic abbreviations are consistent with those in Table 1.

**TABLE 3:** Effect of PAβN on antimicrobial susceptibility of *K. aerogenes^a^*

Antimicrobial agents	MICs (mg/L)						
	R1-PAβN	R2-PAβN	R3-PAβN	R4-PAβN	R5-PAβN	R6-PAβN	R7-PAβN
MEM	≤0.06(S)	≤0.06(S)	≤0.06(S)	≤0.06(S)	8(R)	4(R)	8(R)
IPM	0.125(S)	0.25(S)	0.25(S)	0.5(S)	8(R)	4(R)	8(R)
IMR	≤0.06/4(S)	≤0.06/4(S)	≤0.06/4(S)	≤0.06/4(S)	1/4(S)	2/4(I)	2/4(I)
CAZ	1(S)	2(S)	1(S)	1(S)	2(S)	2(S)	2(S)
CZA	≤0.06/4(S)	≤0.06/4(S)	≤0.06/4(S)	≤0.06/4(S)	2/4(S)	2/4(S)	2/4(S)
ATM	≤0.06(S)	≤0.06(S)	≤0.06(S)	≤0.06(S)	1(S)	1(S)	1(S)
AZA	≤0.06/4(S)	≤0.06/4(S)	≤0.06/4(S)	0.06/4(S)	1/4(S)	1/4(S)	1/4(S)
AMK	2(S)	1(S)	1(S)	2(S)	4(S)	1(S)	1(S)
FEP	≤0.25(S)	≤0.25(S)	≤0.25(S)	≤0.25(S)	16(R)	16(R)	32(R)
ERV	0.125(S)	0.125(S)	0.125(S)	0.125(S)	0.5(S)	0.125(S)	0.25(S)
CRO	≤0.25(S)	≤0.25(S)	≤0.25(S)	≤0.25(S)	32(R)	8(R)	16(R)
CIP	≤0.06(S)	≤0.06(S)	≤0.06(S)	≤0.06(S)	1(R)	1(R)	1(R)
TZP	≤2/4(S)	2/4(S)	≤2/4(S)	4/4(S)	64/4(R)	32/4(R)	32/4(R)
TGC	0.5(S)	0.5(S)	0.5(S)	0.5(S)	2(S)	2(S)	2(S)
COL	0.06(S)	0.06(S)	0.125(S)	0.125(S)	0.125(S)	0.06(S)	0.06(S)

^*a*^ R1-PAβN, R2-PAβN, R3-PAβN, R4-PAβN, R5-PAβN, R6-PAβN, and R7-PAβN represent strains treated with the efflux pump inhibitor PAβN. The full names corresponding to the antibiotic abbreviations are consistent with those in Table 1.

### Analysis of porins in *K. aerogenes*

**Fig 2:**
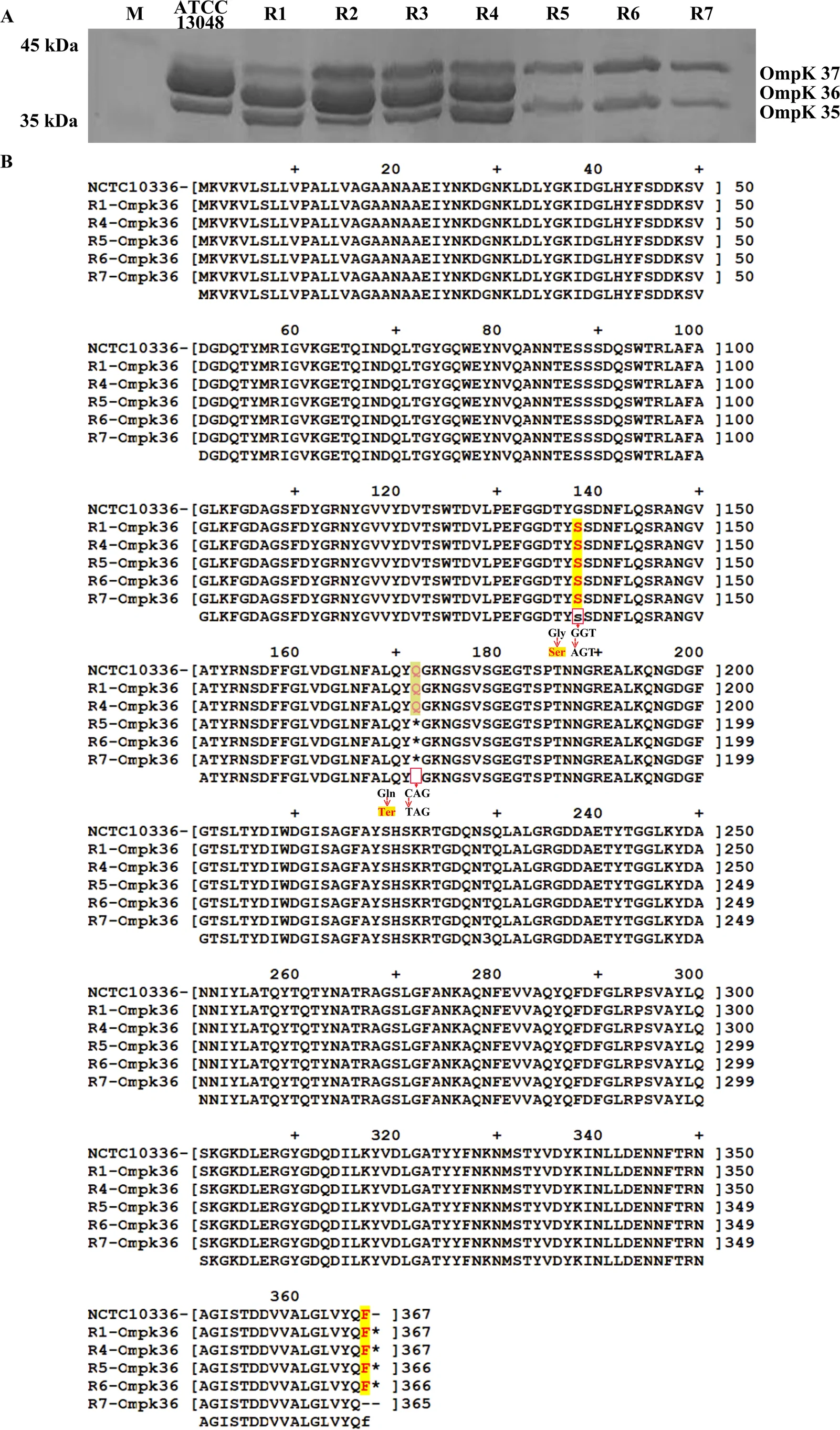
Analysis of the porin in *K. aerogenes*. (**A**) SDS-PAGE analysis of porin expression levels in seven *K. aerogenes* strains. M: protein molecular weight marker; ATCC 13048 served as the control strain. (**B**) Diagram of amino acid sequence alignment for the OmpK36 protein from seven *K. aerogenes* strains.

This study further examined the expression of porins in seven *K. aerogenes* strains. SDS-PAGE results showed that strains R1–R4 expressed both OmpK35 and OmpK36 proteins, with expression patterns similar to the *K. aerogenes* reference strain ATCC 13048. In contrast, the carbapenem-resistant strains R5, R6, and R7 showed expression of only OmpK35 protein, with no detectable OmpK36 protein, suggesting that the absence of OmpK36 expression may be associated with their resistance phenotype (Fig. 2). Further analysis of the amino acid sequences encoded by the *ompK36* gene in clinical isolates revealed that all strains harbored a glycine (Gly) to serine (Ser) substitution at position 138 (corresponding to nucleotide site 412G > A). More importantly, in the carbapenem-resistant strains R5, R6, and R7, an additional C > T mutation at position 514 was identified. This mutation caused the amino acid at position 172 to change from glutamine (Gln) to a termination codon (Ter), forming a prematurely terminated truncated protein (Fig. 2).

### Structural and functional analysis of OmpK36 protein mutations

**Fig 3:**
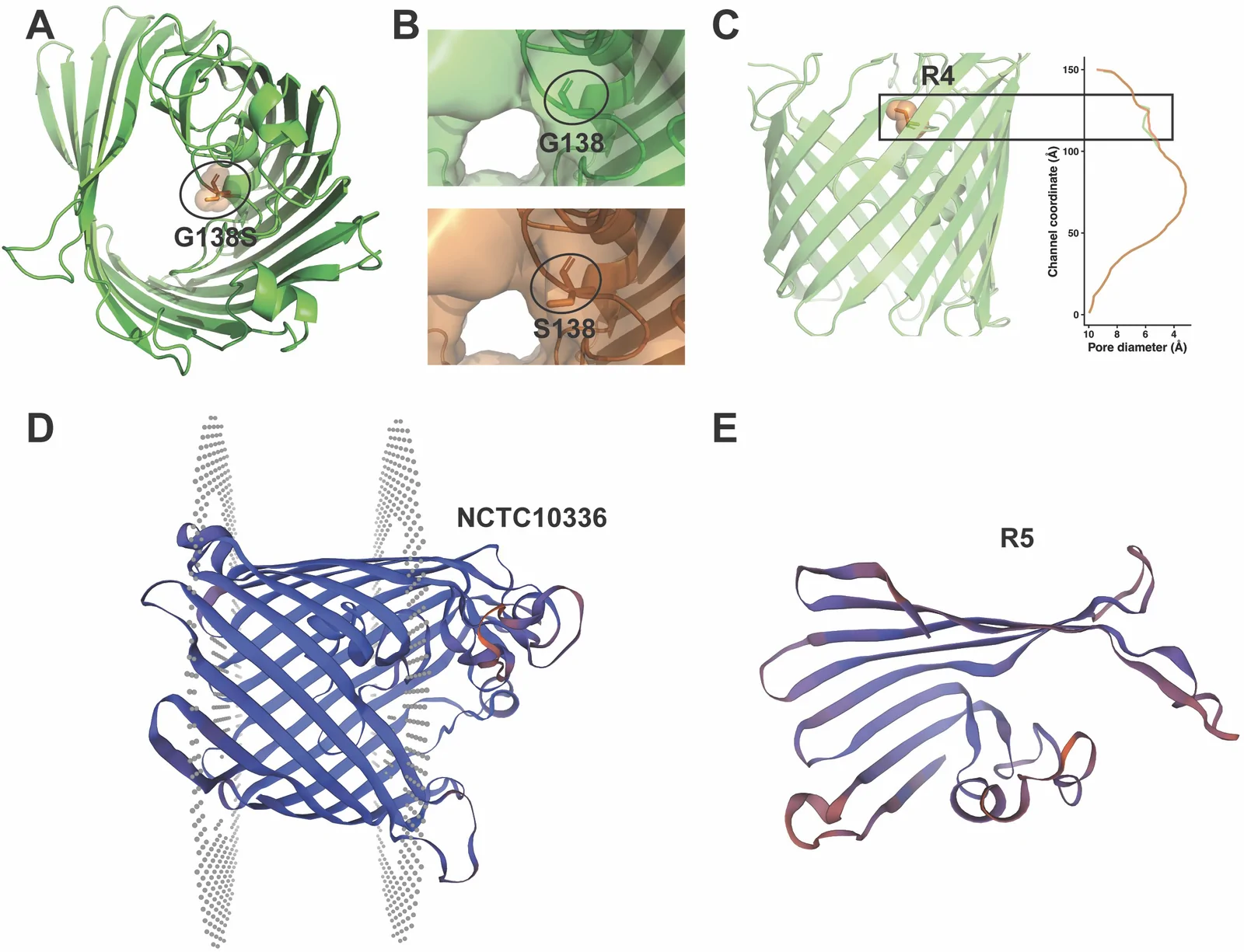
OmpK36 protein mutations cause pore narrowing and structural disruption. (**A**) OmpK36 protein structure showing amino acid position 138 in the control strain (NCTC10336, G138, green) and the mutant strain (R4, S138, orange). (**B**) Side view of the pore surface (gray transparent) and key residues (G138/S138). (**C**) Hole algorithm visualization of pore diameters in OmpK36 proteins from the R4 strain (G138S mutation) and the control strain. (**D**) Schematic of stable anchoring of full-length OmpK36 protein within a phospholipid bilayer. (**E**) Schematic structure of truncated OmpK36 protein (R5, Q172Ter mutation).

To clarify the functional significance of the porin mutation site, this study employed HotSpot Wizard prediction model analysis, revealing that glycine at position 138 of the OmpK36 protein is located in the fifth tunnel (tunnel5, length 36.1 Å, bottleneck radius 1.5 Å) and belongs to pathogenic hotspot amino acids (ranked fifth) (Fig. S2A). Further homology modeling of the OmpK36 protein was performed using Swiss-model, with NTCT10038 as the template (Fig. 3A). Three models were generated: the control NTCT10038, R4, and the carbapenem-resistant R5 strain. The results showed that glycine at position 138 site of the OmpK36 protein is located at the pore channel of the porin, and its mutation alters the pore dimensions (Fig. 3B). Using the Hole algorithm to measure changes in pore size, the study further demonstrated that the amino acid substitution from glycine to serine at position 138 in the OmpK36 protein of the R4 strain reduced the pore size by 15%. This narrowing of the pore is likely to significantly decrease the rate and efficiency of drug passage through the tunnel (Fig. 3C).

The OmpK36 protein contains five tunnels, with the amino acids forming these tunnels predominantly located after position 171 (Table S1). The full-length OmpK36 protein possesses sufficient length to stably anchor to phospholipid bilayers (Fig. 3D). However, in the carbapenem-resistant strain R5, OmpK36 is prematurely truncated at position 172. The truncated protein lacks structural integrity and fails to properly bind ions, such as magnesium, that facilitate drug uptake (Fig. S2B), severely compromising both the structure and function of the porin (Fig. 3D and E).

RMSD analysis based on molecular dynamics simulations showed that the backbone RMSD of NTCT10338 fluctuated minimally and reached equilibrium more rapidly, whereas the backbone and ligand atoms of R5 exhibited higher and more sustained RMSD fluctuations, indicating that both its structure and ligand-binding stability are inferior to those of the full-length protein (NTCT10338) (Fig S3A). Root mean square fluctuation (RMSF) analysis further revealed that the full-length OmpK36 protein displayed overall low fluctuations, while R5 (the truncated protein) showed multiple pronounced fluctuation peaks (RMSF > 12.5 Å) within the residue range 20–120, suggesting that truncation leads to marked structural destabilization in this region, which may impair its channel architecture and function (Fig. S3B).

### *K. aerogenes* produces inducible AmpC β-lactamase

**Fig 4:**
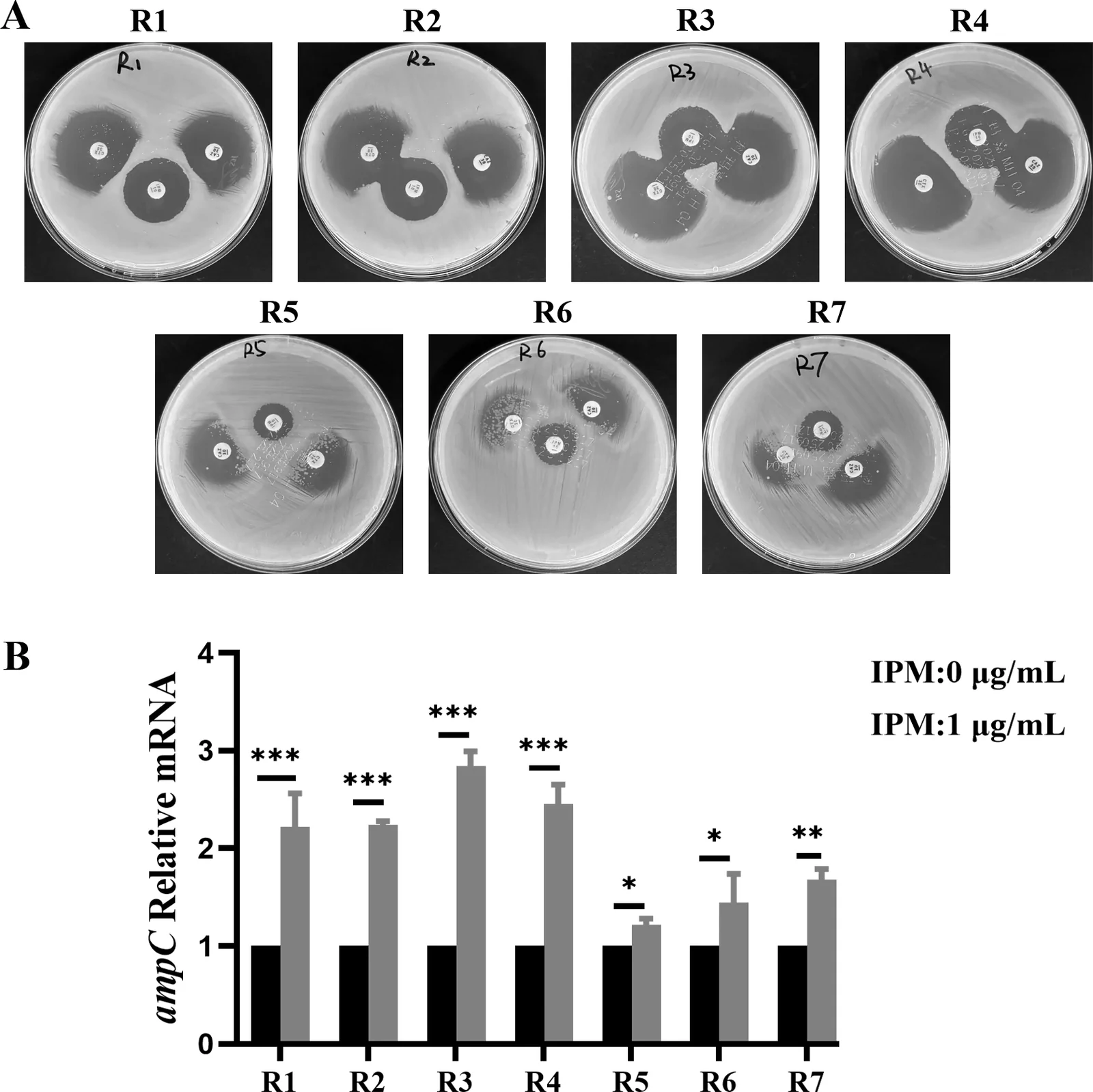
Induction of AmpC enzyme in *K. aerogenes* clinical isolates. (**A**) An AmpC enzyme induction assay was performed using the double-disk synergy test. Bacterial suspensions at 0.5 McFarland standard were spread onto Mueller-Hinton (MH) agar plates. An imipenem (IPM) disk was placed in the center as the inducer, with ceftazidime (CAZ) and cefotaxime (CTX) disks positioned 15 mm away from it. (**B**) Relative *ampC* mRNA levels measured by qPCR in the presence of imipenem. The inducing concentration of imipenem (1 μg/mL) was determined as the highest concentration allowing growth of all tested strains in macrodilution broth, based on a preliminary assay using 1/2 MIC, 1/4 MIC, MIC, 2 MIC, and 4 MIC of imipenem. *P < 0.05; **P < 0.01; ***P < 0.001.

In antimicrobial susceptibility testing, a characteristic “D-shaped inhibition zone” was observed around the ceftazidime-avibactam disks adjacent to imipenem disks for all seven *K. aerogenes* strains (Fig. S1A). To further investigate the resistance mechanisms of these strains, we employed the double-disk synergy test to detect inducible AmpC enzyme expression. The results showed that when imipenem (Fig. 4A) or cefoxitin (Fig. S4) was used as the inducer, all seven clinical strains (R1–R7) displayed typical “D-shaped inhibition zones” around the ceftazidime and cefotaxime discs, indicating that these strains can express inducible AmpC enzymes upon exposure to β-lactam antibiotics. Imipenem is a weak inducer of AmpC enzymes. To verify the above phenotypic observations at the molecular level, we used qPCR to examine the transcriptional levels of the *ampC* gene before and after imipenem stimulation. The results demonstrated that in the presence of imipenem, *ampC* mRNA expression levels in all seven clinical isolates (R1–R7) were significantly higher than those in the uninduced control group, confirming that imipenem induces upregulation of *ampC* gene expression in these strains (Fig. 4B). Notably, the fold induction was lower in the resistant strains (R5–R7) compared with the susceptible strains (R1–R4), which may be attributed to reduced imipenem influx due to OmpK36 deficiency in the resistant strains.

## DISCUSSION

In this study, seven *K. aerogenes* strains were consecutively isolated from bile and ascites fluid of the same patient. Following treatment with imipenem, three subsequent ascites isolates evolved into carbapenem-resistant *K. aerogenes*. These three CRKA strains exhibited atypical carbapenemase phenotypes: resistant to carbapenems, ceftriaxone, and cefpirome, yet susceptible to ceftazidime, with negative results in carbapenemase test, suggesting that the resistance mechanism is not dependent on acquired carbapenemases. Through antimicrobial susceptibility testing, whole-genome sequencing analysis, and AmpC enzyme induction assays, this work revealed a dual-mechanism adaptive evolution under imipenem selection pressure: first, all strains carried the OmpK36 G138S mutation leading to reduced pore diameter, while the resistant strains additionally acquired a Q172Ter truncating mutation, resulting in complete loss of the OmpK36 porin and significantly decreased outer membrane permeability to antibiotics; second, all strains harbored an AmpC enzyme that could be induced by imipenem; although the resistant strains exhibited a diminished induction signal due to porin deficiency, upregulation of *ampC* expression remained detectable. This synergistic effect of the “permeability barrier” and “induced enzyme hydrolysis” collectively led to clinical failure of carbapenem therapy. The elucidation of this specific mechanism provides a new insight and theoretical foundation for the recognition and treatment of similar atypical resistant phenotypes in clinical practice.

A study performed genetic typing on 1,153 clinically isolated *K. aerogenes* strains from China, revealing 366 STs and 24 KL types, including numerous rare sequences and cross-species transfer of capsular types. This fully demonstrates the high diversity of this species in molecular and capsular typing (12). Against this background, all seven strains in this study were confirmed as belonging to ST88 and capsular serotype KL186, with minimal SNP differences among strains, indicating a clonal origin. Currently, reports on the antimicrobial resistance and virulence profiles of ST88 Enterobacterales remain scarce. Notably, a previously reported ST88 *Escherichia coli* strain isolated from blood exhibited resistance to 15 antibiotics across 8 classes, including β-lactams, aminoglycosides, and sulfonamides, while harboring 84 resistance genes, demonstrating an exceptionally robust resistance background (13). However, the ST88 *K. aerogenes* strain in this study exhibits a distinct pathogenic evolutionary pathway: initial infection shows widespread antibiotic susceptibility, but undergoes marked phenotypic resistance shifts under the selective pressure of imipenem therapy (14).

Efflux pumps are protein complexes located on the bacterial cell membrane that actively export a variety of antibiotics, reducing the effective intracellular drug concentration and thereby mediating resistance (15, 16). Whole-genome sequencing revealed efflux pump-related gene mutations in all seven *K. aerogenes* strains. To evaluate their role in resistance, this study conducted antimicrobial susceptibility testing using efflux pump inhibitors. Results showed reduced MIC values for certain drugs (e.g., ceftriaxone) but no fundamental shift in resistance/susceptibility patterns. Further comparison indicated that the carbapenem-resistant strains shared identical efflux pump-related gene sequences with the susceptible strains, revealing no novel mutations. This finding demonstrates that efflux pumps are not the primary mechanism driving resistance in these strains.

Porins are channel proteins on the outer membrane of Gram-negative bacteria, which exhibit reduced expression, deletion, or structural mutations, leading to decreased outer membrane permeability and preventing antibiotics (such as β-lactams and fluoroquinolones) from entering the bacterial interior (17, 18). SDA-PAGE analysis of *K. aerogenes* revealed that OmpK36 protein was undetectable in all carbapenem-resistant strains (R5, R6, R7), in stark contrast to susceptible strains. Sequence alignment of the OmpK36 gene showed that at amino acid position 138, glycine (G) was substituted by serine (S) in all seven *K. aerogenes* strains. Homology modeling and channel-pore analysis indicated that this mutation lies in a key region of the pore constriction zone and reduces the pore diameter by approximately 15%. This implies that even in susceptible strains, the permeation rate of drug molecules through the OmpK36 protein has already decreased (19). However, this reduction in pore size alone is insufficient to confer a carbapenem resistance phenotype, suggesting that this degree of decreased permeability has not yet crossed the resistance threshold.

In carbapenem-resistant strains, another key mutation was identified: a mutation of glutamine to a stop codon at position 172 of the OmpK36 gene (Q172Ter), leading to premature termination of protein translation and the production of a truncated OmpK36 protein. From a structural biology perspective, the impact of a premature termination mutation on pore function depends on the region where the truncation occurs. If the truncation occurs in the pore-forming region, it could lead to abnormal channel structure, potentially even forming a larger pore diameter that might conversely facilitate drug diffusion. However, in this study, Q172Ter is located in the C-terminal anchoring domain, a region responsible for the correct folding of the protein and its anchoring to outer membrane lipopolysaccharides. The truncated protein, due to the loss of the C-terminal domain, cannot be correctly localized to the outer membrane nor form a complete transmembrane channel. At this point, the concept of a “pore diameter” no longer applies; instead, there is a complete absence of functional porin. Therefore, although a truncation mutation could theoretically pose a risk of increased pore size, the actual consequence here, due to its specific location, is the complete disappearance of the channel. For the resistant strain, not only do narrowed channels exist on its outer membrane due to the G138S mutation (which is still present in the resistant strain’s genome), but more importantly, due to the Q172Ter truncation mutation, functional OmpK36 protein is completely absent. This creates an almost insurmountable permeability barrier against carbapenems, conferring the resistance phenotype upon the strain.

AmpC enzyme, primarily produced by Gram-negative bacteria, is an inducible enzyme whose expression level depends on the binding status of the AmpR regulator with cell-wall peptidoglycan degradation products (20). Although *ampC* genes are commonly carried on the chromosome of *K. aerogenes*, the presence of an inducible phenotype varies among strains (21). In this study, all seven clinical isolates exhibited upregulation of *ampC* gene expression upon imipenem stimulation, and phenotypic induction assays confirmed the presence of D-shaped inhibition zones upon exposure to β-lactam agents, indicating that these strains possess the characteristic of inducible AmpC enzyme production. Notably, the fold induction of *ampC* was lower in the resistant strains (R5–R7) compared with the susceptible strains (R1–R4). This difference may be attributed to the complete loss of OmpK36 porin in the resistant strains: owing to porin deficiency, the amount of imipenem entering the cells is significantly reduced, leading to a weaker induction signal in the periplasmic space and consequently a relatively lower level of induced *ampC* gene expression. Nonetheless, significant upregulation of *ampC* was still detectable in the resistant strains, suggesting that even a small amount of drug entry is sufficient to trigger the induction signal.

Although the susceptible strains R1–R4 also carry inducible AmpC enzymes and harbor the OmpK36 G138S substitution that results in a narrowed pore diameter, the OmpK36 protein in these strains remains intact without truncating mutations, thereby retaining partial channel function and allowing a certain amount of drug entry. Consequently, this feature alone is insufficient to confer carbapenem resistance. In the resistant strains, the inducible AmpC enzyme and the complete loss of the porin OmpK36 together constitute a synergistic resistance mechanism. During imipenem treatment, the absence of OmpK36 serves as the first physical barrier that impedes drug entry, while the small amount of imipenem that does enter acts as an induction signal to stimulate AmpC enzyme expression. Thus, the minimal amount of drug that manages to penetrate the outer membrane barrier is likely hydrolyzed by the induced AmpC enzyme in the periplasmic space. This dual effect—restricted drug entry coupled with intracellular drug inactivation—ultimately confers the carbapenem-resistant phenotype in the resistant strains.

This study systematically reveals that a group of clinically derived ST88 *K. aerogenes* strains evolved into pathogens possessing both virulence and multidrug-resistant traits through a composite resistance mechanism centered on structural distortion and loss of the porin OmpK36 coupled with inducible AmpC β-lactamase production. These findings deepen our understanding of carbapenem resistance mechanisms that are not mediated by carbapenemases. Inducible AmpC is likely an important yet frequently overlooked contributor to carbapenem treatment failure. The phenotypic detection method for such inducible enzymes employed in this study is straightforward and easy to implement. Incorporating this type of inducibility testing into the routine laboratory work-up of *K. aerogenes*, especially as a routine auxiliary identification for strains showing poor response to β-lactam therapy—holds urgent practical value for accurate early warning of resistance risks and for optimizing clinical antimicrobial strategies.

## MATERIALS AND METHODS

### Bacterial strains and antimicrobial susceptibility testing

Seven *K. aerogenes* strains (designated *K. aerogenes* R1–R7) were isolated from a 77-year-old male patient hospitalized at Jiaxing Second Hospital. All strains were identified as *K. aerogenes* using the VITEK-2 compact system with MALDI-TOF mass spectrometry (BioMérieux, France). Antimicrobial susceptibility testing was performed by the broth microdilution method. MIC results for all tested agents except tigecycline and aztreonam-avibactam were interpreted according to the Clinical and Laboratory Standards Institute 2025 breakpoints. Tigecycline MICs were interpreted using U.S. Food and Drug Administration (FDA) criteria (susceptible ≤ 2 mg/L, intermediate 4–8 mg/L, resistant ≥ 8 mg/L). Aztreonam-avibactam MICs were interpreted based on FDA breakpoints (susceptible ≤ 4/4 mg/L, intermediate 8/4 mg/L, resistant ≥ 16/4 mg/L). *Escherichia coli* ATCC 25922 was used as the quality control strain for susceptibility testing.

### Whole-genome sequencing and bioinformatics analysis

Genomic DNA was extracted from five *K. aerogenes* isolates using a commercial DNA extraction kit (TIANGEN, Beijing, China) according to the manufacturer’s instructions. All strains were subjected to next-generation sequencing on the Illumina NovaSeq platform (Illumina Inc.) with a 150-bp paired-end strategy. The resulting sequencing reads were assembled *de novo* using SPAdes software (v3.15.5) (22). Antimicrobial resistance genes and virulence factors were identified using the ResFinder database (v4.1) and the Virulence Factor Database, respectively (23, 24). Multilocus sequence typing (MLST) was determined according to the *K. aerogenes* scheme on the PubMLST website (https://pubmlst.org/). To analyze variations in the key porin OmpK36, the *ompK36* gene and its encoded amino acid sequences were aligned and phylogenetically examined using MEGA software (24). Based on the wild-type sequence, homology modeling of the OmpK36 protein was performed via the Swiss-Model server (https://swissmodel.expasy.org/) (25), and the HOLE algorithm (26) was further employed to calculate and compare the transmembrane pore radii of different mutant protein models. SNP analysis was conducted using the Snippy pipeline (version 4.6.0, https://github.com/tseemann/snippy). Whole-genome annotation was carried out with ProKKa (v1.14.6) (27). The whole-genome sequencing data of the *K. aerogenes* isolates have been deposited in the NCBI GenBank database under the following accession numbers: R1: JBVYHU000000000, R4: JBVYOG000000000, R5: JBVYHT000000000, R6: JBVYHL000000000, and R7: JBVYOH000000000. These data are associated with BioProject accession numbers PRJNA1433197 (R1), PRJNA1433213 (R4), PRJNA1433221 (R5), PRJNA1433222 (R6), and PRJNA1433212 (R7).

### Efflux pump inhibitor test

CCCP, a proton ionophore, inactivates efflux pumps dependent on proton motive force by disrupting the proton gradient across the bacterial inner membrane. PAβN dihydrochloride acts as a competitive inhibitor, interacting with the substrate-binding sites of RND family efflux pumps, thereby blocking antibiotic efflux. Stock solutions of CCCP and PaβN (MedChemExpress LLC, USA) were prepared by dissolving 10 mg of each powder in 7.71 and 7.72 mL of DMSO (Sigma, USA), respectively, to achieve a concentration of 1,280 μg/mL. CCCP and PAβN were then diluted in Mueller-Hinton broth to final concentrations of 5 and 25 μg/mL, respectively, and added to the test bacterial suspension adjusted to a 0.5 McFarland standard. Subsequent procedures were performed in accordance with routine antimicrobial susceptibility testing. Interpretation criteria: a ≥4-fold reduction in the MIC of an antibiotic in the presence of an efflux pump inhibitor, compared to the control without the inhibitor, indicates that resistance to the antibiotic in the tested strain is associated with an active efflux mechanism.

### Inducible AmpC β-lactamase testing

The phenotypic detection of inducible AmpC enzyme was performed using the double-disk synergy test (28). Briefly, three single colonies of *K. aerogenes* were suspended in 0.9% saline and adjusted to a 0.5 McFarland standard. The suspension was evenly swabbed onto a Mueller-Hinton agar plate. An AmpC-inducer disk (cefoxitin or imipenem) was placed at the center of the plate, and indicator disks (cefotaxime and ceftazidime) were symmetrically positioned 15 mm away from the inducer disk. After incubation at 37°C for 12–16 h, the plates were examined. A positive result was defined as a flattening or indentation of the inhibition zone of any indicator disk on the side facing the inducer disk, forming a “Keyhole” or “D-shaped” synergistic effect zone, indicating the presence of an inducible AmpC enzyme. If all inhibition zones remained round and without distortion, the result was interpreted as negative (29).

### Extraction of porins and SDS-PAGE analysis

*K. aerogenes* ATCC13048 and strains R1–R7 were cultured overnight in Mueller-Hinton broth at 37°C with shaking at 220 rpm in a constant temperature shaker (HerryTech Co., Ltd., Shanghai, China). Bacteria were harvested by centrifugation at 12,000 rpm for 10 min into 2 mL sterile EP tubes. The cell pellets were washed with 750 μL of PBS buffer (Yeasen Biotechnology, China), centrifuged at 12,000 rpm for 10 min, and the supernatant was discarded. This washing step was repeated once. The pellet was then resuspended in 750 μL of PBS. Following cell lysis using a sonicator, the lysate was centrifuged at 15,600 × *g* for 1 h at 4°C. The pellet was resuspended in 400 μL of PBS buffer containing 2% (wt/vol) Sarcosyl (BBI Life Sciences, China) and incubated at room temperature for 30 min (with vortexing every 5 min). The mixture was then centrifuged at 15,600 × *g* for 1 h at 4°C, and the supernatant was discarded. The resulting pellet, containing the porins, was resuspended in 50 μL of PBS.

A 10 μL 5× protein loading buffer (Beyotime Biotech Inc., China) was added to 40 μL of the porins sample, and the mixture was denatured by heating at 100°C for 10 min. Subsequently, 10 μL of each sample was loaded into the wells of a 12% polyacrylamide separating gel (Yeasen Biotechnology, China) for SDS-PAGE protein separation at 110 V. Electrophoresis was terminated when the protein samples reached the bottom of the separating gel. The gel was then immersed in BeyoBlue Plus Coomassie Blue Super Fast Staining Solution (Beyotime Biotech Inc., China) at room temperature for 30 min, followed by washing with ddH₂O 3–5 times to remove excess stain and visualize the major protein bands. *K. aerogenes* 13048 (a strain known to express OmpK35 and OmpK36) served as the control strain for porins.

### Real-time reverse transcription-polymerase chain reaction

Real-time reverse transcription-polymerase chain reaction was employed to detect the gene expression levels of strains R1–R7. For RNA extraction, single colonies of the strains were inoculated in Mueller-Hinton broth and cultured overnight at 37°C with shaking at 220 rpm in a constant temperature incubator. Total RNA was extracted using the MolPure Bacterial RNA Kit (Yeasen Biotechnology, China), following the manufacturer’s instructions. RNA was reverse transcribed into cDNA using the Hifair AdvanceFast One-step RT-gDNA Digestion SuperMix (Yeasen Biotechnology, China). The reaction system comprised: 1 μg of total RNA, 5 μL of 4× Hifair AdvanceFast One-Step RT SuperMix, and 1 μL of gDNA Remover Mix, made up to a final volume of 20 μL with RNase-free water. The reaction conditions were 37°C for 5 min, followed by 85°C for 30 s. The cDNA concentration was determined using a Qubit fluorometer (Thermo Fisher Scientific, USA), and the cDNA was subsequently diluted to a concentration of 100 ng/μL. Gene expression levels were measured by quantitative real-time PCR using Hieff UNICON Advanced qPCR SYBR Master Mix. The reaction system consisted of 10 μL of Hieff UNICON Advanced qPCR SYBR Master Mix, 0.4 μL of Forward Primer (10 μM), 0.4 μL of Reverse Primer (10 μM), 2 μL of cDNA, and 7.2 μL of ddH₂O. The thermal cycling conditions were initial denaturation at 95°C for 2 min, followed by 40 cycles of denaturation at 95°C for 10 s and annealing/extension at 60°C for 30 s. A melting curve analysis was performed using the instrument’s default program. Gene expression levels were normalized to the 16s rRNA gene and calculated using the 2^−ΔΔCT^ method based on the Ct values. The primer sequences used were *16s*-F (5′-TACCGCATAACGTCGCAAGA-3′), *16s*-R (5′-GGACCGTGTCTCAGTTCCAG-3′), *ampC*-F (5′-AGGACTACGCCTGGGGTTAT-3′), and *ampC*-R (5′-CGCTGTTCATATTCGCCAGC-3′). All experiments were independently repeated three times. Data are presented as mean ± standard deviation (SD). Comparisons between two groups were performed using an unpaired two-tailed Student’s *t*-test. Statistical analyses and graphical representations were conducted using GraphPad Prism version 9.

### Carbapenemase detection

Carbapenemase inhibition enhancement test: three single colonies of *K. aerogenes* R5–R7 were collected using a cotton swab and suspended in 0.9% saline to prepare a 0.5 McFarland standard bacterial suspension. The suspension was evenly streaked onto a Mueller-Hinton (MH) agar plate. Four imipenem discs with different compositions were placed on each plate: an imipenem disc alone (bacterial resistance control), an imipenem disc supplemented with 5 μL of boronic acid, an imipenem disc supplemented with 5 μL of EDTA, and an imipenem disc supplemented with 5 μL of boronic acid and 5 μL of EDTA. The plates were incubated at 37°C for 16–24 h before observing the results. Interpretation criteria: when the inhibition zone diameter of the imipenem disc supplemented with boronic acid or EDTA was ≥5 mm larger than that of the imipenem disc alone, the isolate was interpreted as producing a class A or class B carbapenemase, respectively. When the inhibition zone diameter of the imipenem disc supplemented with both boronic acid and EDTA was ≥5 mm larger than that of the imipenem disc alone, the isolate was interpreted as producing both class A and class B carbapenemases (note: in routine practice, a ceftazidime-avibactam disc is also placed on the same plate to provide guidance for clinical medication).

Colloidal gold immunochromatographic assay: the carbapenemase types of *K. aerogenes* R5–R7 were detected using a carbapenemase detection kit (Gold Mountain River, China) according to the manufacturer’s instructions.

## Supplementary Material

Reviewer comments

## SUPPLEMENTAL MATERIAL

Supplemental material spectrum.04108-25-s0001.pdf Fig. S1 to S4; Table S1.

OPEN PEER REVIEW reviewer-comments.pdf An accounting of the reviewer comments and feedback.

ASM does not own the copyrights to Supplemental Material that may be linked to, or accessed through, an article. The authors have granted ASM a non-exclusive, world-wide license to publish the Supplemental Material files. Please contact the corresponding author directly for reuse.
