# Supplementary material for: OmpK36 deficiency and inducible AmpC β-lactamase synergistically drive imipenem resistance in Klebsiella aerogenes
Source: Microbiol Spectr. 2026 May 29;14(7):e04108-25. doi: 10.1128/spectrum.04108-25 (PMC13340039; doi:10.1128/spectrum.04108-25)
Supplement: Supplemental material — Fig. S1 to S4; Table S1. [file spectrum.04108-25-s0001.pdf]

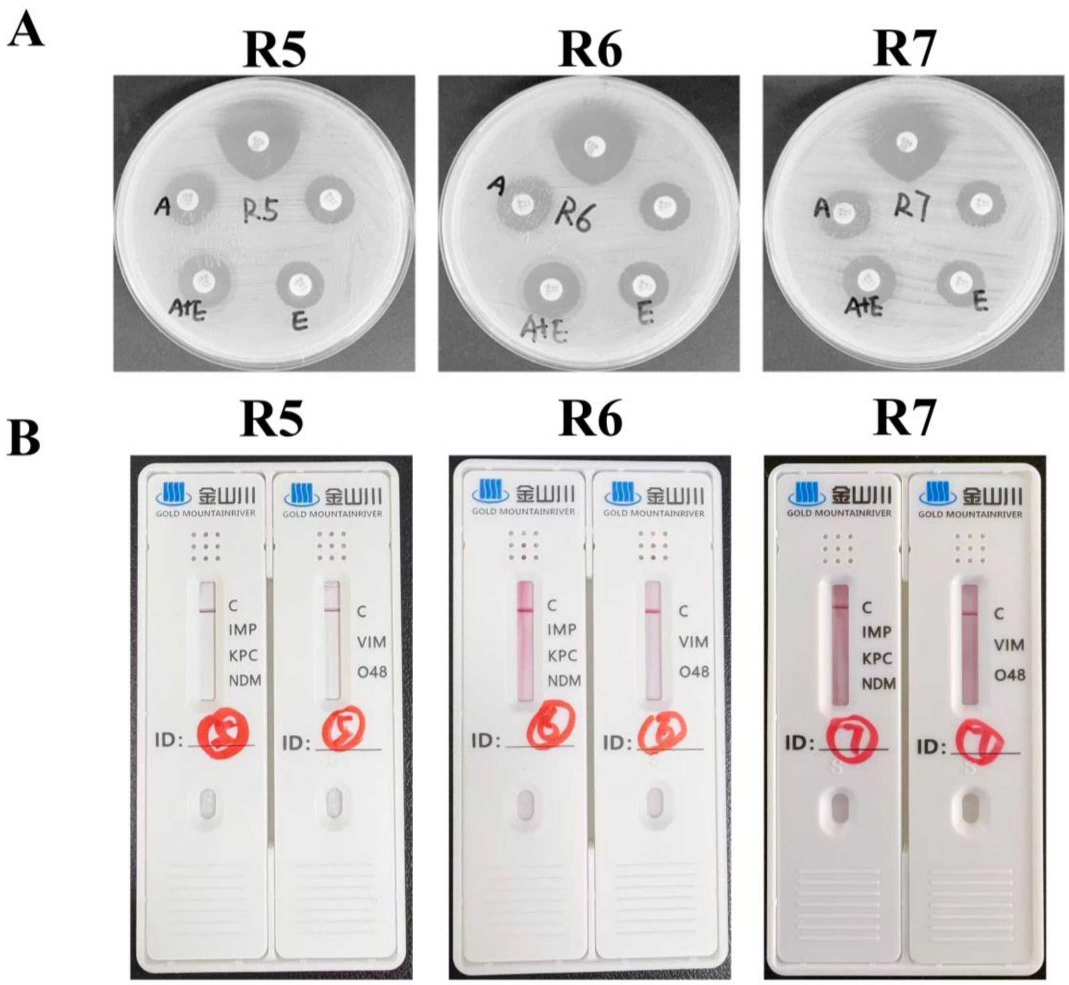


**FIG S1** Phenotypic detection of carbapenemases in *K.aerogenes*. (A) Detection of carbapenemase phenotype in CRKA strains using the carbapenemase inhibition enhancement test(The text on the plate: A indicates the disc supplemented with boric acid, E indicates the disc supplemented with EDTA, and A+E indicates the disc supplemented with both boric acid and EDTA.); (B) Detection of carbapenemase phenotype in CRKA strains using the colloidal gold method.

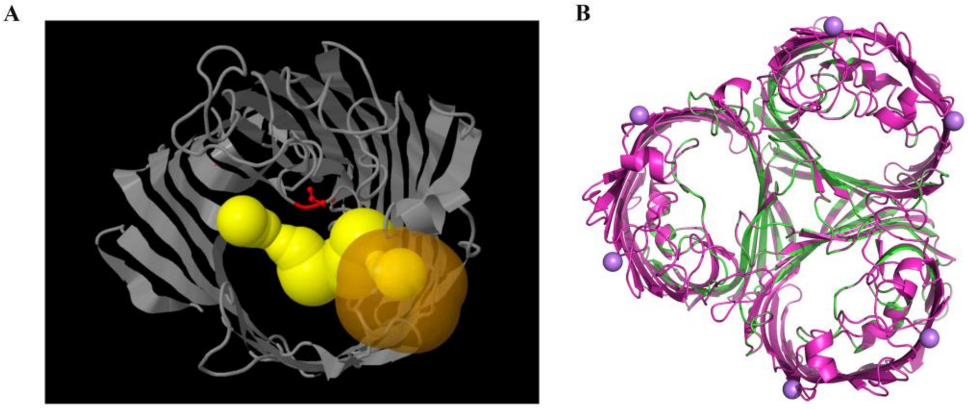

**FIG S2** Genetic mutations in the OmpK36 protein lead to structural and functional defects. (A) Tunnel architecture of the OmpK36 protein predicted using the HotSpot Wizard model. The glycine at position 138 (highlighted in red ball-and-stick representation) is located within the fifth tunnel (tunnel5, length 36.1 Å, bottleneck radius 1.5 Å) and was predicted to be a pathogenic hotspot amino acid (ranked fifth). (B) Structural superimposition of the truncated OmpK36 protein from the R5 strain (pink) and the wild-type OmpK36 protein from the NCTC 10336 strain (green). The truncated protein in the R5 strain loses its structural integrity, resulting in an inability to properly bind ions such as magnesium that facilitate drug uptake.

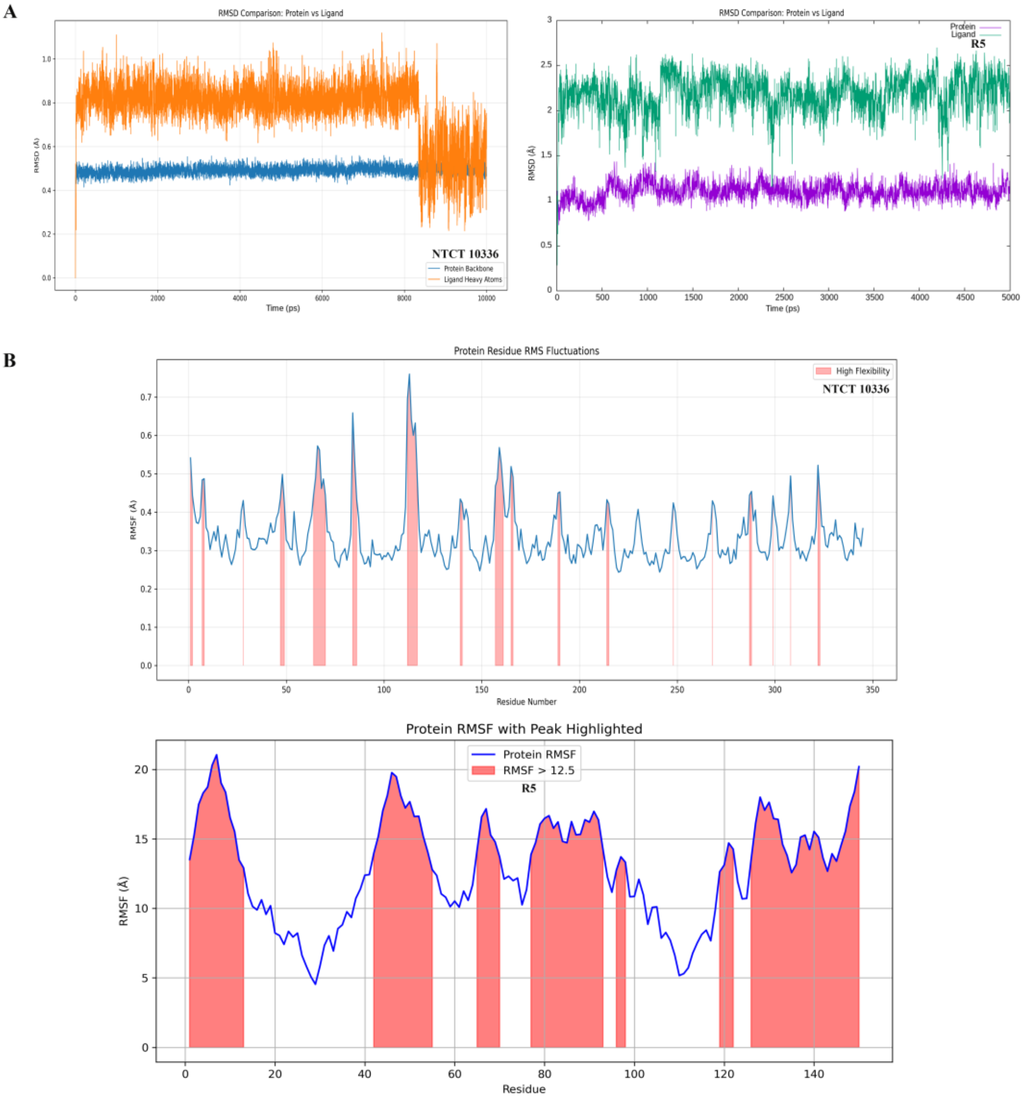


**FIG S3** Molecular dynamics simulation analysis reveals structural stability defects in the truncated OmpK36 protein from the R5 strain. (A) Root-mean-square deviation (RMSD) analysis from molecular dynamics simulations comparing the truncated OmpK36 mutant (R5) and the wild-type OmpK36 protein.(B) Root-mean-square fluctuation (RMSF) analysis

from molecular dynamics simulations comparing the truncated OmpK36 mutant (R5) and the wild-type OmpK36 protein.

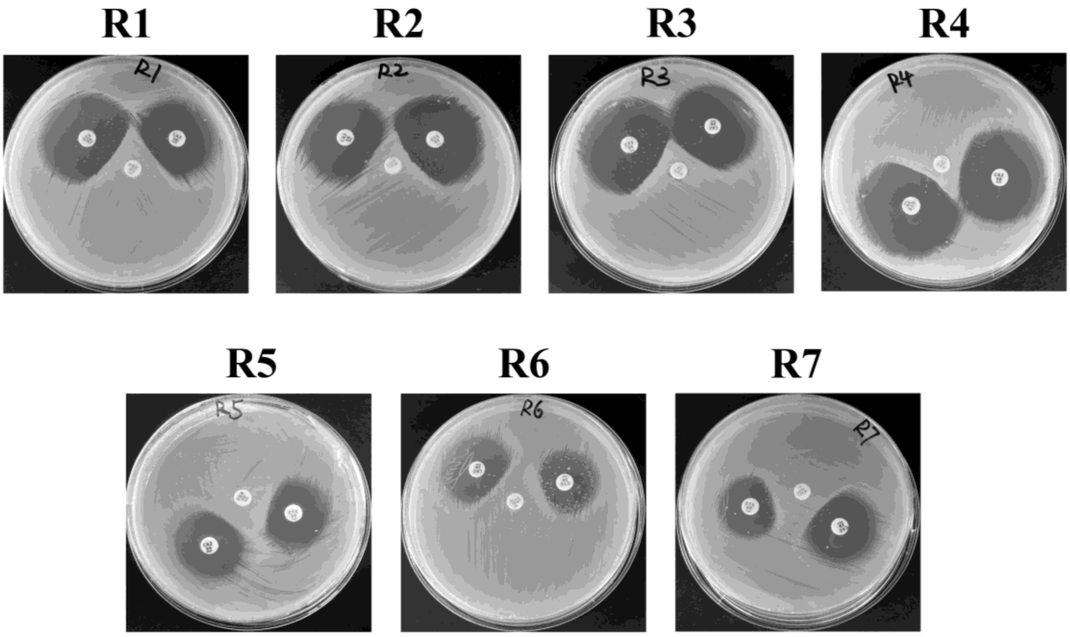


**FIG S4** Results of the AmpC enzyme induction test (cefoxitin as inducer). R1–R7 are consecutive *K. aerogenes* isolates obtained from the same patient.

**Table S1**. Architecture and amino acid composition of tunnels in the OmpK36 protein predicted by HotSpot Wizard.

| Tunne | Length(Å) | bottleneck radius(Å) | Amino acid composition | | | | |
|---|---|---|---|---|---|---|---|
| 1 | 3.4 | 2.9 | 47 Asp | 349 Arg | 356 Asp | | |
| | | | 48 Lys | 351 Ala | | | |
| | | | 49 Ser | 352 Gly | | | |
| | | | 50 Val | 353 Ile | | | |
| | | | 344 Glu | 354 Ser | | | |
| | | | 348 Thr | 355 Thr | | | |
| 2 | 8.3 | 2.4 | 46 Asp | 345 Asn | 353 Ile | | |
| | | | 47 Asp | 348 Thr | 354 Ser | | |
| | | | 48 Lys | 349 Arg | 355 Thr | | |
| | | | 49 Ser | 350 Asn | 356 Asp | | |
| | | | 50 Val | 351 Ala | | | |
| | | | 344 Glu | 352 Gly | | | |
| 3 | 7.5 | 1.8 | 47 Asp | 348 Thr | 355 Thr | | |
| | | | 48 Lys | 349 Arg | 356 Asp | | |
| | | | 49 Ser | 351 Ala | | | |
| | | | 50 Val | 352 Gly | | | |
| | | | 338 Lys | 353 Ile | | | |
| | | | 344 Glu | 354 Ser | | | |
| 4 | 29.4 | 1.5 | 37 Lys | 80 Asn | 135 Asp | 334 Tyr | 356 Asp |
| | | | 39 Asp | 96 Arg | 136 Thr | 338 Lys | 358 Val |
| | | | 41 Leu | 116 Tyr | 137 Tyr | 340 Asn | 366 Gln |
| | | | 43 Tyr | 120 Tyr | 138 Gly | 342 Leu | |
| | | | 47 Asp | 127 Asp | 146 Arg | 344 Glu | |
| | | | 48 Lys | 128 Val | 267 Arg | 348 Thr | |
| | | | 49 Ser | 129 Leu | 268 Ala | 349 Arg | |
| | | | 50 Val | 130 Pro | 269 Gly | 351 Ala | |
| | | | 54 Gln | 131 Glu | 270 Ser | 352 Gly | |
| | | | 56 Tyr | 132 Phe | 315 Ile | 353 Ile | |
| | | | 58 Arg | 133 Gly | 316 Leu | 354 Ser | |
| | | | 78 Glu | 134 Gly | 318 Tyr | 355 Thr | |
| 5 | 36.1 | 1.5 | 41 Leu | 95 Thr | 140 Asp | 338 Lys | 358 Val |
| | | | 43 Tyr | 96 Arg | 146 Arg | 340 Asn | |
| | | | 47 Asp | 114 Arg | 148 Asn | 342 Leu | |
| | | | 49 Ser | 131 Glu | 190 Arg | 344 Glu | |
| | | | 50 Val | 132 Phe | 194 Lys | 348 Thr | |
| | | | 54 Gln | 133 Gly | 267 Arg | 349 Arg | |
| | | | 56 Tyr | 134 Gly | 268 Ala | 351 Ala | |
| | | | 58 Arg | 135 Asp | 269 Gly | 352 Gly | |
| | | | 80 Asn | 136 Thr | 270 Ser | 353 Ile | |
| | | | 82 Gln | 137 Tyr | 315 Ile | 354 Ser | |
| | | | 93 Ser | 138 Gly | 316 Leu | 355 Thr | |
| | | | 94 Trp | 139 Ser | 318 Tyr | 356 Asp | |
