# Supplementary material for: OmpK36 deficiency and inducible AmpC β-lactamase synergistically drive imipenem resistance in Klebsiella aerogenes
Source: Microbiol Spectr. 2026 May 29;14(7):e04108-25. doi: 10.1128/spectrum.04108-25 (PMC13340039; doi:10.1128/spectrum.04108-25)
Supplement: Reviewer comments [file reviewer-comments.pdf]

AMERICAN SOCIETY FOR MICROBIOLOGY | Microbiology Spectrum

OmpK36 deficiency and inducible AmpC β-lactamase synergistically drive imipenem resistance in *Klebsiella aerogenes*

Shumi Shang, Liyang Chen, xiaosi li, Siqi Xu, heping shen, Fuping HU, Wenting Tang, and xiaoyan wu

Corresponding Author(s): xiaoyan wu, The Second Affiliated Hospital of Jiaxing University

Review Timeline:

Submission Date:	December 24, 2025
Editorial Decision:	January 30, 2026
Revision Received:	March 21, 2026
Accepted:	April 13, 2026

Editor: Gregory Wiedman

Reviewer(s): Disclosure of reviewer identity is with reference to reviewer comments included in decision letter(s). The following individuals involved in review of your submission have agreed to reveal their identity: Shankar Manoharan (Reviewer #1)

Transaction Report:

(Note: With the exception of the correction of typographical or spelling errors that could be a source of ambiguity, letters and reports are not edited. The original formatting of letters and referee reports may not be reflected in this compilation.)

DOI: https://doi.org/10.1128/spectrum.04108-25

Re: Spectrum04108-25 (**OmpK36 deficiency and inducible AmpC β-lactamase synergistically drive imipenem resistance in *Klebsiella aerogenes***)

Dear Ms. Wenting Tang:

Thank you for the privilege of reviewing your work. Below you will find my comments, instructions from the Spectrum editorial office, and the reviewer comments.

Please return the manuscript within 60 days; if you cannot complete the modification within this time period, please contact me. If you do not wish to modify the manuscript and prefer to submit it to another journal, notify me immediately so that the manuscript may be formally withdrawn from consideration by Spectrum.

Revision Guidelines
To submit your modified manuscript, log into the submission site at https://spectrum.msubmit.net/cgi-bin/main.plex. Go to Author Tasks and click the appropriate manuscript title to begin. The information you entered when you first submitted the paper will be displayed; update this as necessary. Note the following requirements:

• Upload point-by-point responses to the issues raised by the reviewers in a file named "Response to Reviewers," NOT in your cover letter.
• Upload a compare copy of the manuscript (without figures) as a "Marked-Up Manuscript" file.
• Upload a clean .DOC/.DOCX version of the revised manuscript and remove the previous version.
• Each figure must be uploaded as a separate, editable, high-resolution file (TIFF or EPS preferred), and any multipanel figures must be assembled into one file.
• Any supplemental material intended for posting by ASM should be uploaded with their legends separate from the main manuscript. You can combine all supplemental material into one file (preferred) or split it into a maximum of 10 files with all associated legends included.

For complete guidelines on revision requirements, see our Submission and Review Process webpage. Submission of a paper that does not conform to guidelines may delay acceptance of your manuscript.

Data availability: ASM policy requires that data be available to the public upon online posting of the article, so please verify all links to sequence records, if present, and make sure that each number retrieves the full record of the data. If a new accession number is not linked or a link is broken, provide Spectrum production staff with the correct URL for the record. If the accession numbers for new data are not publicly accessible before the expected online posting of the article, publication may be delayed; please contact production staff (Spectrum@asmusa.org) immediately with the expected release date.

Publication Fees: For information on publication fees and which article types are subject to charges, visit our website. If your manuscript is accepted for publication and any fees apply, you will be contacted separately about payment during the production process; please follow the instructions in that e-mail. Arrangements for payment must be made before your article is published.

ASM Membership: Corresponding authors may join or renew ASM membership to obtain discounts on publication fees. Need to upgrade your membership level? Please contact Customer Service at Service@asmusa.org.

The ASM Journals program strives for constant improvement in our submission and publication process. Please tell us how we can improve your experience by taking this quick Author Survey.

Thank you for submitting your paper to Spectrum.

Sincerely,
Gregory Wiedman
Editor
Microbiology Spectrum

Reviewer #1 (Comments for the Author):

The study by Shang and colleagues aims to characterize a series of Klebsiella aerogenes isolates recovered from a patient with a post-pancreaticoduodenectomy infection. The initial isolates were sensitive to carbapenems. The infection was treated with imipenem, after which carbapenem-resistant K. aerogenes (R5, R6, R7) was isolated from the patient's ascites. However, the isolates were found not to produce any carbapenemases. The study aims to elucidate the mechanism underlying the emergence

of carbapenem resistance in K. aerogenes. Using efflux pump inhibitors, the authors show that drug efflux only minimally contributes to the carbapenem resistance phenotype seen. In a disc diffusion assay, the disk bearing ceftazidime-avibactam showed a D-shaped zone of inhibition facing the disk bearing imipenem, suggestive of an imipenem-inducible beta-lactamase active against ceftazidime-avibactam. The authors propose that the inducible beta-lactamase is AmpC. Further, the authors identified a G138S substitution in the OmpK36 porin in isolates R1 and R4 (carbapenem-sensitive) and in isolates R5, R6, and R7 (carbapenem-resistant). The resistant isolates R5, R6 and R7 also harboured a premature stop codon at position 172, resulting in a truncated OmpK36 protein. Structure prediction analysis of the OmpK36 porin with and without the G138S substitution was carried out. The authors claim that the substitution narrows the pore. Considering these two findings, the major conclusion of the study is that carbapenem resistance results from: 1) reduced drug permeability due to loss of OmpK36 and 2) hydrolysis by the inducible beta-lactamase. The article leverages available patient isolates and aims to explain the emergence of carbapenem resistance by combining phenotyping and genome sequencing. However, I have the following concerns that need to be considered and addressed:

Major concerns:
• The structure prediction studies suggest a decrease in the pore size of the OmpK36 G138S variant. However, this does not seem relevant, as the substitution is also detected in the sensitive strains (R1 and R4). These strains also harbour an inducible beta-lactamase but remain carbapenem-susceptible.
• The authors claim that OmpK36 was undetectable in strains R5, R6, and R7. Given that the SDS-PAGE profiles of the strains R5, R6, and R7 have very high background relative to the other isolates analyzed. Can this be confirmed by Western blotting?
• The materials and methods section is quite brief and is not detailed enough to allow replication of the findings. For example, there is no information on how the outer membrane proteins were prepared for SDS-PAGE.
• While the characteristic D-shaped inhibition zones do point towards an inducible beta-lactamase. The study does not provide evidence that the inducible beta-lactamase is indeed AmpC.
• It is unclear if the genome sequence data of the isolates have been deposited in a repository, where they can be accessed. Accession numbers pointing to publicly available sequence data must be made available. This is important because most of the study's conclusions are based on genome sequence data.

Minor concerns:
• Even if minimally, drug efflux also seems to contribute to carbapenem resistance in the resistant isolates. Could the authors explain this phenomenon from the genome sequence data?
• Ln 129: The genetic loci for aerobactin are incorrectly indicated as acrA and acrB (efflux pump encoding genes). This should be revised to the iucABCD-iutA locus.
• Ln 131: Gene name for ompA is incorrectly written as OmpA. The manuscript may be proofread to eliminate such errors.
• The authors indicate that carbapenemase inhibitor enhancement test and colloidal gold assay for carbapenemases were carried out to rule out the acquisition of carbapenemases. This data can be shown. Alternatively, it may be indicated that the data is not shown.

Reviewer #2 (Comments for the Author):

The manuscript from Shang, et al explores the progression of a clinical isolate of K. aerogenes from carbapenem susceptible to carbapenem resistant without the acquisition of a carbapenemase. Studies show that the isolate develop mutations in the OmpK36 porin over the course of treatment, in addition to the presence of inducible AmpC. The manuscript presents a comprehensive analysis of a phenomenon that is becoming more common in clinical settings of carbapenem resistance in the absence of a detectable carbapenemase. There are a few points that could be clarified to strengthen the submission.

1. On lines 115-119 there is discussion of a D-zone in the presence of ceftaz-avi plus imipenem in the results section. This is not depicted in figure 4 or the supplemental materials, so think this needs to be revised.
2. Additionally, the same thing is referenced in line 194=195 with an indication to refer to supplemental figure 3a. That does not include imipenem as the inducer or ceftazidime avibactam.
3. The induction experiments should be addressed in a single section of the results. It is unclear to me why the control strains were chosen (particularly ones that include fully resistant isolates). Since cefoxitin and imipenem are known inducers of ampC and the control strains in S3a are not showing a "D" zone, is the imipenem induction significant? Could this be a product of another resistance mechanism.
4. Lastly, on the note of the AmpC, line 217 concludes it is persistently activated which also calls into question the induction. Please consider a revision of this set of experiments and conclusion.
5. For table 2 it might be beneficial to narrow down to representative strains before and after resistance was acquired. It was difficult to review with the volume of data in the table. But I didn't notice any striking differences from 1-4 to 5-7.
6. The discussion around the virulence seems like it could be excluded or shortened to summarize that other virulence associated genes remained unchanged over the course of the clinical case.
7. The abstract states that the isolate remained susceptible to ceftaz/avi which is a bit misleading since the isolate remained susceptible to ceftazidime alone.
8. Please review the spacing in the manuscript, often missing space between sentences or words in the discussion. Line 212,

line 271, line 273 as examples.

Dear Editor and Reviewers:

We sincerely thank you for reviewing our manuscript entitled "OmpK36 deficiency and inducible AmpC β-lactamase synergistically drive imipenem resistance in *Klebsiella aerogenes*" and for your valuable comments. We have carefully considered each comment and made corresponding revisions to the manuscript. The changes have been highlighted in purple in the text, and our point-by-point responses are provided below (in blue).

Reviewer #1 (Comments for the Author):

The study by Shang and colleagues aims to characterize a series of Klebsiella aerogenes isolates recovered from a patient with a post-pancreaticoduodenectomy infection. The initial isolates were sensitive to carbapenems. The infection was treated with imipenem, after which carbapenem-resistant K. aerogenes (R5, R6, R7) was isolated from the patient's ascites. However, the isolates were found not to produce any carbapenemases. The study aims to elucidate the mechanism underlying the emergence of carbapenem resistance in K. aerogenes. Using efflux pump inhibitors, the authors show that drug efflux only minimally contributes to the carbapenem resistance phenotype seen. In a disc diffusion assay, the disk bearing ceftazidime-avibactam showed a D-shaped zone of inhibition facing the disk bearing imipenem, suggestive of an imipenem-inducible beta-lactamase active against ceftazidime-avibactam. The authors propose that the inducible beta-lactamase is AmpC. Further, the authors identified a G138S substitution in the OmpK36 porin in isolates R1 and R4 (carbapenem-sensitive) and in isolates R5, R6, and R7 (carbapenem-resistant). The resistant isolates R5, R6 and R7 also harboured a premature stop codon at position 172, resulting in a truncated OmpK36 protein. Structure prediction analysis of the OmpK36 porin with and without the G138S substitution was carried out. The authors claim that the substitution narrows the pore. Considering these two findings, the major conclusion of the study is that carbapenem resistance results from: 1) reduced drug permeability due to loss of OmpK36 and 2) hydrolysis by the inducible beta-lactamase. The article leverages available patient isolates and aims to explain the emergence of carbapenem resistance by combining phenotyping and genome sequencing. However, I have the following concerns that need to be considered and addressed:

Major concerns:

• The structure prediction studies suggest a decrease in the pore size of the OmpK36 G138S variant. However, this does not seem relevant, as the substitution is also detected in the sensitive strains (R1 and R4). These strains also harbour an inducible beta-lactamase but remain carbapenem-susceptible.

Response： Thank you for your valuable comments. Your query regarding the presence of the G138S mutation in susceptible strains and its association with resistance is highly precise, and we have systematically revised the relevant discussion in the revised manuscript. When we initially discovered that R5-R7 were resistant while carbapenemase testing was negative, whole-genome sequencing alignment revealed two mutations in OmpK36: G138S (shared by all strains) and Q172Ter (unique to resistant strains). Given that OmpK36 is a porin protein, mutations or truncations may affect channel size, but it was not possible to determine whether these mutations would enlarge or narrow the pore. Therefore, we conducted homology modeling to calculate the pore diameter of the protein. However, as you correctly pointed out, susceptible strains also carry

this mutation yet remain susceptible, indicating that pore narrowing alone is insufficient to confer resistance. In resistant strains, the Q172Ter truncation mutation results in loss of the C-terminal anchoring domain, preventing proper localization of the protein in the outer membrane—structural simulations indicate that this truncated protein cannot stably embed into the phospholipid bilayer, consistent with the complete absence of OmpK36 protein in resistant strains as detected by SDS-PAGE. Therefore, the essential characteristic of resistant strains is 'complete porin loss against a background of channel narrowing', whereas susceptible strains, despite having narrowed channels, retain residual porin proteins that maintain basal permeability. Regarding inducible AmpC enzyme, we observed D-shaped zones in enzyme inhibition enhancement tests, and qPCR confirmed significant upregulation of *ampC* expression in all seven strains following imipenem induction. Interestingly, both susceptible and resistant strains exhibited this characteristic. Although susceptible strains possess the inducible enzyme and channel narrowing, the absence of complete porin loss means they do not yet cross the resistance threshold; in resistant strains, complete channel loss synergizes with the inducible enzyme to ultimately confer the resistance phenotype. Based on your comments, we have clearly distinguished between the different functional levels of 'pore narrowing' and 'complete channel loss' in the abstract and discussion, and have marked the revised paragraphs in purple in the manuscript (lines 19–31, 262–284). Thank you again for your guidance.

• The authors claim that OmpK36 was undetectable in strains R5, R6, and R7. Given that the SDS-PAGE profiles of the strains R5, R6, and R7 have very high background relative to the other isolates analyzed. Can this be confirmed by Western blotting?

Response: Thank you for your thorough review and valuable comments. Regarding this issue, we have optimized the experiment: we re-prepared outer membrane protein samples, quantified the total protein concentration using the BCA method, and ensured equal loading across all lanes to eliminate any interference from loading differences in band observation. After repeating the SDS-PAGE under optimized conditions (see revised Figure 2A), the results still clearly show the absence of protein bands at the molecular weight corresponding to OmpK36 in strains R5, R6, and R7, while distinct bands are visible in strains R1-R4 and the reference strain ATCC13048. We agree that Western blot is a more specific detection method. However, commercially available OmpK36-specific antibodies are currently difficult to obtain, and antibodies targeting this protein in *K.aerogenes* are not yet widely available, posing practical challenges for conducting Western blot. It should be noted that whole-genome sequencing in this study has confirmed the presence of the Q172Ter truncation mutation in resistant strains, which leads to premature termination of protein translation, providing strong corroborating evidence from the genetic level for the protein absence observed in SDS-PAGE. The genetic evidence and the optimized protein electrophoresis results corroborate each other, collectively supporting the conclusion of OmpK36 protein loss in resistant strains.

• The materials and methods section is quite brief and is not detailed enough to allow replication of the findings. For example, there is no information on how the outer membrane proteins were prepared for SDS-PAGE.

Response: Thank you for your correction. Following your suggestions, we have comprehensively supplemented and refined the Materials and Methods section to ensure the reproducibility of the experimental procedures. The specific modifications can be found in the corresponding sections of the manuscript (lines 353-367, 380-447). Thank you again for your valuable comments.

• While the characteristic D-shaped inhibition zones do point towards an inducible beta-lactamase. The study does not provide evidence that the inducible beta-lactamase is indeed AmpC.

Response: Thank you for your correction. In response to this issue, we have added evidence for the identification of AmpC enzyme in the revised manuscript: (1) At the molecular level, qPCR analysis revealed that *ampC* gene expression levels in seven clinical strains were significantly higher after imipenem induction than in the uninduced control group(Figure. 4B); (2) The inhibitor potentiation test and colloidal gold method showed that all strains were negative for carbapenemases (Figure. S1). Together with whole-genome sequencing, which did not detect any other β-lactamase genes (e.g., bla_{KPC}, bla_{NDM}, bla_{OXA}), this excluded interference from other enzyme types; (3) The double-disk synergy test itself is a classic phenotypic method for detecting inducible AmpC enzyme. In the Materials and Methods section, we have cited two classic references to further clarify the methodological basis(Reference 28). These multiple lines of evidence corroborate each other and collectively support the conclusion that the inducible enzyme carried by the strains in this study is AmpC.

• It is unclear if the genome sequence data of the isolates have been deposited in a repository, where they can be accessed. Accession numbers pointing to publicly available sequence data must be made available. This is important because most of the study's conclusions are based on genome sequence data.

Response: Thank you for kindly pointing out this issue. We have uploaded the whole-genome sequencing data of the seven strains to the NCBI database and have supplemented the corresponding sequence accession numbers in the Materials and Methods section (line353-358).

Minor concerns:

• Even if minimally, drug efflux also seems to contribute to carbapenem resistance in the resistant isolates. Could the authors explain this phenomenon from the genome sequence data?

Response: Thank you for your question. We fully agree that efflux pumps represent one of the important mechanisms of bacterial drug resistance. In this study, we conducted efflux pump inhibition assays using CCCP and PAβN. The results showed that the MICs of the tested antibiotics did not decrease significantly upon the addition of the inhibitors. According to current interpretation criteria, this suggests that efflux pumps are not the primary mechanism contributing to carbapenem resistance in the strains studied. Additionally, analysis of the whole-genome sequencing data revealed no acquired mutations in known major efflux pump genes or significant variations in their regulatory elements. Based on this combined phenotypic and genotypic evidence, we conclude that efflux mechanisms do not play a key role in the resistance phenotype observed in this study, and thus, this aspect was not discussed in depth in the manuscript.

• Ln 129: The genetic loci for aerobactin are incorrectly indicated as acrA and acrB (efflux pump encoding genes). This should be revised to the iucABCD-iutA locus.

Response: Thank you very much for pointing out this non-standard part in our paper. We have revised the corresponding section in the manuscript accordingly(line 126,128,131).

• Ln 131: Gene name for ompA is incorrectly written as OmpA. The manuscript may be proofread to eliminate such errors.

Response: Thank you very much for raising this concern. We have carefully consulted the nomenclature rules for bacterial gene names and revised the entire manuscript accordingly; the revisions have been highlighted in purple in the text.

• The authors indicate that carbapenemase inhibitor enhancement test and colloidal gold assay for

carbapenemases were carried out to rule out the acquisition of carbapenemases. This data can be shown. Alternatively, it may be indicated that the data is not shown.

Response: Thank you for your suggestion. We have supplemented the results of the carbapenemase inhibitor enhancement test and the colloidal gold method in Supplementary Figure 1 and have indicated these additions at the corresponding locations in the main text. The revisions have been highlighted in purple for your review(Figure S1).

Reviewer #2 (Comments for the Author):

The manuscript from Shang, et al explores the progression of a clinical isolate of K. aerogenes from carbapenem susceptible to carbapenem resistant without the acquisition of a carbapenemase. Studies show that the isolate develop mutations in the OmpK36 porin over the course of treatment, in addition to the presence of inducible AmpC. The manuscript presents a comprehensive analysis of a phenomenon that is becoming more common in clinical settings of carbapenem resistance in the absence of a detectable carbapenemase. There are a few points that could be clarified to strengthen the submission.

1. On lines 115-119 there is discussion of a D-zone in the presence of ceftaz-avi plus imipenem in the results section. This is not depicted in figure 4 or the supplemental materials, so think this needs to be revised.

Response: Thank you for your suggestion. We have supplemented the results of the carbapenemase inhibitor enhancement test and the colloidal gold method in Supplementary Figure 1. The revisions have been highlighted in purple in the text for your review(line115).

2. Additionally, the same thing is referenced in line 194=195 with an indication to refer to supplemental figure 3a. That does not include imipenem as the inducer or ceftazidime avibactam.

Response: Thank you for your suggestion. We have carefully revised the manuscript and supplemented the results of the carbapenemase inhibitor enhancement test and the colloidal gold method in Supplementary Figure 1. The revisions have been highlighted in purple in the text for your review(line192).

3. The induction experiments should be addressed in a single section of the results. It is unclear to me why the control strains were chosen (particularly ones that include fully resistant isolates). Since cefoxitin and imipenem are known inducers of ampC and the control strains in S3a are not showing a "D" zone, is the imipenem induction significant? Could this be a product of another resistance mechanism.

Response: Thank you for your question. The double-disc synergy test in this study was originally designed to explain the phenomenon of a D-shaped inhibition zone around the ceftazidime-avibactam disc adjacent to the imipenem disc observed in the enzyme inhibitor enhancement test, thereby elucidating the mechanistic basis of resistance in the strains. In *Enterobacterales*, genes encoding AmpC enzymes are ubiquitously present on the chromosome, but under basal conditions most strains express extremely low levels of the enzyme, and the small amount produced can be recycled by the cell wall. Upon induction by β-lactam agents, AmpC enzymes can be substantially overexpressed; however, whether they are ultimately released into the periplasmic space to exert

hydrolytic activity depends on the strain's cell wall recycling capacity and the balance of its regulatory system (for example, in some strains, even though AmpC enzymes expression is elevated, the enzyme may still be effectively recycled by the cell wall). In addition, plasmid-borne *ampC* genes (such as bla_{DHA} and bla_{CMY} types) can also lead to constitutive hyperproduction. To further validate the inducible nature, we performed qPCR and found that in the reference strain ATCC13048, *ampC* expression also increased following imipenem stimulation, yet no D-shaped inhibition zone was observed; similarly, the three bile-derived *K. aerogenes* strains with different susceptibility phenotypes selected for this study also did not exhibit this phenotype. Given the lack of genomic data for these control strains, we are unable to provide a definitive explanation for their negative phenotypes, although this observation warrants further investigation. To avoid potential ambiguity introduced by the control strains and to maintain a more focused scope, we have removed the results pertaining to the control strains in the revised manuscript, concentrating solely on the mechanistic characterization of the seven strains themselves. Thank you again for your valuable comments(Figure 4, line192-205).

4. Lastly, on the note of the AmpC, line 217 concludes it is persistently activated which also calls into question the induction. Please consider a revision of this set of experiments and conclusion.

Response: Thank you for your suggestion. We have systematically revised the conclusions regarding AmpC based on your comments. We have supplemented the qPCR experiment, which confirmed that *ampC* expression was significantly increased in all seven strains following imipenem induction; accordingly, the conclusion originally described as "constitutive activation" has been revised to "inducible expression with release into the periplasmic space." Corresponding adjustments have been made in the Abstract and Discussion sections of the manuscript. Thank you again for your guidance(Figure4, lines 19–31, 262–284).

5. For table 2 it might be beneficial to narrow down to representative strains before and after resistance was acquired. It was difficult to review with the volume of data in the table. But I didn't notice any striking differences from 1-4 to 5-7.

Response: Thank you for your suggestion. We have split the original Table 2 into two separate tables (now Table 2 and Table 3) to present the susceptibility results for the efflux pump inhibitors CCCP and PAβN, respectively, thereby improving the clarity of data presentation. Regarding your suggestion to present only representative strains, we have decided not to adopt this approach for now, as susceptibility results typically need to be presented in full to ensure data reproducibility; however, we have enhanced the readability of the data by splitting the tables.

6. The discussion around the virulence seems like it could be excluded or shortened to summarize that other virulence associated genes remained unchanged over the course of the clinical case.

Response: Thank you for your suggestion. Based on your comments, we have streamlined the discussion on virulence factors, retaining only a summary statement that they remain stable during the clinical course(line 234-237).

7. The abstract states that the isolate remained susceptible to ceftaz/avi which is a bit misleading since the isolate remained susceptible to ceftazidime alone.

Response: Thank you for your correction. Based on your comments, we have revised the statement regarding ceftazidime-avibactam in the Abstract to avoid any potential misinterpretation. The specific revisions can be found in the Abstract section(line19-31).

8. Please review the spacing in the manuscript, often missing space between sentences or words in the discussion. Line 212, line 271, line 273 as examples.

Response: Thank you for pointing out the formatting oversights in the manuscript. We have carefully reviewed the entire text and corrected the missing spaces in the Discussion section (lines 225, 241, etc.). In the future, we will pay closer attention to formatting standards to avoid such errors. Thank you again for your thorough review.

Re: Spectrum04108-25R1 (**OmpK36 deficiency and inducible AmpC β-lactamase synergistically drive imipenem resistance in *Klebsiella aerogenes***)

Dear Prof. xiaoyan wu:

Your manuscript has been accepted, and I am forwarding it to the ASM production staff for publication. Your paper will first be checked to make sure all elements meet the technical requirements. ASM staff will contact you if anything needs to be revised before copyediting and production can begin. Otherwise, you will be notified when your proofs are ready to be viewed.

Data Availability: ASM policy requires that data be available to the public upon online posting of the article, so please verify all links to sequence records, if present, and make sure that each number retrieves the full record of the data. If a new accession number is not linked or a link is broken, provide production staff with the correct URL for the record. If the accession numbers for new data are not publicly accessible before the expected online posting of the article, publication may be delayed; please contact ASM production staff immediately with the expected release date.

Publication Fees: For information on publication fees and which article types have charges, please visit our website. We have partnered with Copyright Clearance Center (CCC) to collect author charges. If fees apply to your paper, you will receive a message from no-reply@copyright.com with further instructions. For questions related to paying charges through RightsLink, please contact CCC at ASM_Support@copyright.com or toll free at +1-877-622-5543. CCC makes every attempt to respond to all emails within 24 hours.

ASM Membership: Corresponding authors may join or renew ASM membership to obtain discounts on publication fees. Need to upgrade your membership level? Please contact Customer Service at Service@asmusa.org.

Embargo Policy: A press release may be issued as soon as the manuscript is posted on the Spectrum Latest Articles webpage. The corresponding author will receive an email with the subject line "ASM Journals Author Services Notification" when the article is available online.

The ASM Journals program strives for constant improvement in our submission and publication process. Please tell us how we can improve your experience by taking this quick Author Survey.

Thank you for submitting your paper to Spectrum.

Sincerely,
Gregory Wiedman
Editor
Microbiology Spectrum
